# Miniaturized Microfluidic Electrochemical Biosensors Powered by Enzymatic Biofuel Cell

**DOI:** 10.3390/bios13020175

**Published:** 2023-01-22

**Authors:** Linlin Wang, Wenlei Zhu, Jianrong Zhang, Jun-Jie Zhu

**Affiliations:** 1 Shaanxi Key Laboratory of Chemical Additives for Industry, College of Chemistry and Chemical Engineering, Shaanxi University of Science and Technology, Xi’an 710021, China; 2 School of Chemistry and Chemical Engineering, School of Environment, State Key Laboratory of Analytical Chemistry for Life Science, Nanjing University, Nanjing 210023, China

**Keywords:** electrochemical biosensor, enzymatic biofuel cell, self-powered biosensor, microfluidic technology, screen-printing

## Abstract

Electrochemical biosensors, in which enzymatic biofuel cells simultaneously work as energy power and signal generators, have become a research hotspot. They display the merits of power self-support, a simplified structure, in vivo operational feasibility, online and timely monitoring, etc. Since the concept of enzymatic biofuel cell-powered biosensors (EBFC-SPBs) was first proposed, its applications in health monitoring have scored tremendous achievements. However, the creation and practical application of portable EBFC-SPBs are still impeded by the difficulty in their miniaturization. In recent years, the booming microfluidic technology has powerfully pushed forward the progress made in miniaturized and portable EBFC-SPBs. This brief review recalls and summarizes the achievements and progress made in miniaturized EBFC-SPBs. In addition, we also discuss the advantages and challenges that microfluidic and screen-printing technologies provide to wearable and disposable EBFC-SPBs.

## 1. Introduction

The concept of EBFC-SPBs was pioneered by Katz in 2001, as he found that the power output of the EBFC depended linearly on concentrations of fuels (glucose and lactate) [1]. Essentially, an EBFC-SPB is a well-designed EBFC with a simple two-electrode structure, and thus it holds great potential for creating portable and miniaturized electrochemical biosensors [2,3,4,5,6]. In particular, the power source of an EBFC-SPB is the energy that the EBFC harvests from the bio-fluids, making EBFC-SPBs ideal online biosensors [7,8,9,10,11]. In addition, as enzymes are highly specific biocatalysts, the EBFC-SPB possesses outstanding anti-interference capability compared to traditional electrochemical biosensors [12,13,14,15,16,17,18]. These advantages of EBFC-SPBs greatly stimulate researchers’ enthusiasm. Efforts to advance the sensing functions enabled EBFC-SPBs to play a major role in the fields of environment monitoring, artificial organs, and disease diagnosis and therapy [7,19,20,21,22,23,24,25]. Significantly, with the growing demand for personalized, portable medical devices, the portability, one of the most distinctive properties of EBFC-SPBs, has attracted tremendous interest [26,27,28,29,30,31,32,33]. The necessary factors for creating portable EBFC-SPBs are to reduce the sensors’ size and improve the sensors’ wearability. 

Nevertheless, miniaturizing EBFC-SPBs will bring new challenges to precisely control fuels, manipulate sensors, and maintain a high signal output and timely response [34,35,36,37,38,39]. What is noteworthy is that microfluidic technology can effectively respond to the above major challenges [36,40,41,42,43]. Moore et al. heralded the era of microfluidic biofuel cells [44]. As a new type of energy supply device, the microfluidic EBFC possesses a miniaturized size, low cost, and efficient power output. In particular, the flow manner of anodic and cathodic fuels in the microchannel is parallel laminar flow, effectively avoiding turbulent mixing. As a result, the fuels in microfluidic EBFC can be precisely controlled. With the increasingly sophisticated nature of microfluidic EBFCs, miniaturized EBFC-SPBs built on microfluidic chip emerged [45,46]. 

In addition to the advantage of electric energy self-sufficiency, microfluidic EBFC-SPBs can be built on functional substrates (such as flexible and lightweight substrates) with millimeter-sized channels using a simple methods [12,19,31,33,34,42]. Considering that the electrode reactions proceed in the millimeter-sized channels, the demand for catalysts and samples decreased, and eventually, the cost of EBFC-SPBs significantly decreased. Additionally, the electrolyte can automatically flow into the millimeter-sized channel due to the capillary action, further simplifying the sensor structure [36,47,48]. Taken together, the small microfluidic EBFC-SPB (μ-EBFC-SPB) shows great application potential in creating disposable, wearable, and portable bio-sensing devices [12,19,20]. Nevertheless, it is a great challenge to modify the materials and catalysts on centimeter-sized or even millimeter-sized electrodes. Significantly, screen-printing technology opens up new prospects for developing functional μ-EBFC-SPBs because this technology can be used to subtly engineer miniaturized electrodes via precisely printing electrode materials or enzyme on the electrodes. 

In this review article, the recent advancements in μ-EBFC-SPBs will be discussed in detail from the aspects of fabrication, function, performance improvement, and design consideration. The application-oriented challenges in future research and development will also be analyzed. 

## 2. Enzymes in the EBFC-SPBs

The enzymes used in EBFC-SPB systems can be divided into two categories according to their redox effects: anodic enzymes and cathodic enzymes. Specifically, bilirubin oxidase (BOD) and laccase are frequently used cathodic enzymes that catalyze the reduction of oxygen in body fluids [2,4]. Considering that glucose and lactate are the two most common biofuels in body fluids, glucose oxidase (GOD), glucose dehydrogenase (GDH), and lactate oxidase are frequently used anodic enzymes. Lactate oxidase is used to catalyze the oxidation of lactate. Both GOD and GDH are used to catalyze the oxidation of glucose. The difference is that GOD is an aerobic dehydrogenase that specifically oxidizes glucose into gluconic acid and hydrogen peroxide while GDH can directly catalyze glucose oxidation without being affected by oxygen but needs a coenzyme as an auxiliary catalyst [6,8]. 

Compared with abiotic catalysts, enzymatic catalysts exhibit more efficient catalytic activity in a mild biological environment. However, as electrocatalysts, these enzymes have intrinsic drawbacks. The redox active sites of enzymes are usually buried deep within their insulating protein matrix, hindering the electron transfer between the active sites and the electrode surface [1,8,39]. Therefore, in order to improve the performance of the EBFC, two approaches are required: selecting appropriate electron mediators to mediate the electron transfer and developing electron-conductive electrode material to shorten the distance between the active sites of the enzyme and electrode surface. Using mediators can accelerate the electron transfer rate, but it will reduce the voltage output of the EBFC [30,35,39]. In addition, as exogenous substances, the usage of mediators can cause potential harm to living bodies. For efficient electrode materials, they must be conductive enough to transfer electrons quickly, be nanoscale to place enzymes closer to the electrode surface, and have a large surface area to increase the enzyme loadings [20,29]. Therefore, we need to choose appropriate enzyme catalysts and electrode preparation strategies according to the practical application of EBFC-SPBs. 

## 3. Disposable Microfluidic EBFC-SPBs

**Figure 1:**
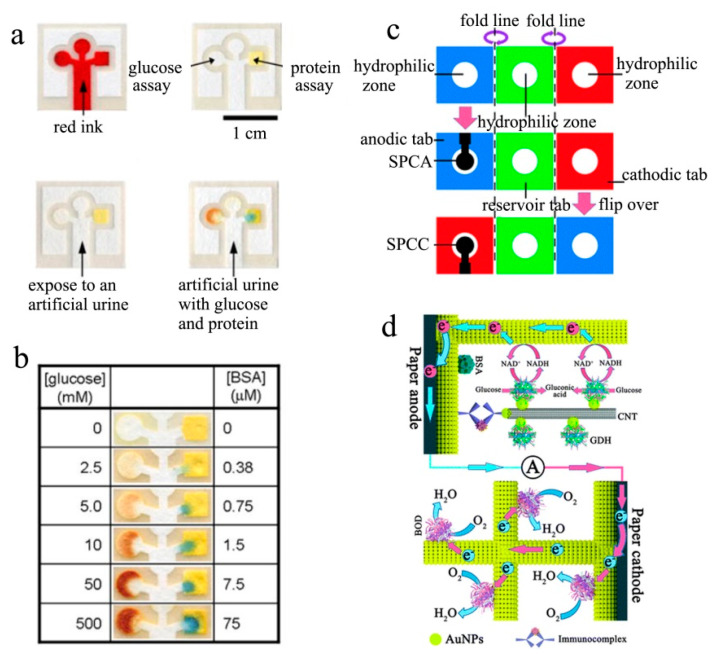
(**a**) Structure diagram and working principle of the chromatography paper-based biosensor. (**b**) The color changes of this paper-based sensor for different concentrations of glucose and BSA [49]. Copyright 2007 John Wiley and Sons. (**c**) Schematic representation and (**d**) working principle of the 3D-m-OBFCAD [50]. Copyright 2014 Royal Society of Chemisty.

The first microfluidic biosensor was pioneered by Whitesides et al. in 2007 [49]. They built an enzyme catalytic biosensor on a well-patterned paper with millimeter-sized channels using the photoetching technique. As Figure 1a,b show, this microfluidic biosensor could simultaneously detect glucose and protein using only 0.3 μL enzyme solutions and 5 μL urine samples. In particular, the capillary action was able to filter out large particles of contaminants, such as dust in the sample, realizing the accurate and anti-interference detection in outdoor contamination conditions. Therefore, this microfluidic biosensor was disposable and portable due to its low cost and miniaturized size, and it was expected to replace expensive clinical diagnostic equipment. However, limited by immature fabrication technology, this sensing system still has several shortcomings: a) the sensing system cannot offer accurate quantitative information of analytes depending on the color changes, b) the response time is too long (over 15 min), and c) the derivatization agents and mediators are required, complicating the device fabrication and operation.

Despite the above drawbacks, the research of Whitesides et al. demonstrated that microfluidic paper-supported biosensors represent an outstanding paradigm for disposable sensing devices. Given this, Ge et al. designed a mediator-free, glucose-air EBFC on microfluidic paper, proposing the first μ-EBFC-SPB for immunoassays [50]. As Figure 1c depicts, this μ-EBFC-SPB was created on a novel three-dimensional microfluidic origami that consisted of three square tabs (1.5 cm × 1.5 cm). In order to ensure an efficient electrical contact between the enzyme catalyst and the electrode, the authors used up-to-scale and economic screen-printed technology to fabricate the Ab1-modified Au anode and bilirubin oxidase (BOD)-modified Au cathode (Figure 1d). The open-circuit voltage and the maximum power density of the resulting μ-EBFC-SPB reached up to 0.85 V and 130.7 mW cm^−2^, respectively, as it was competitive with the conventional glucose–air biofuel cell. In the presence of the target antigen, the signal antibody-modified glucose dehydrogenase could be fixed on the Ab1-Au anode, forming a complete electron path with the BOD cathode. The sensor’s current output responded promptly and linearly to the target concentration. Benefitting from the efficient electricity generation performance, the antigen detection limit was as low as 0.85 pg mL^−1^. The outstanding sensitivity of this μ-EBFC-SPB makes it easier to develop into miniaturized, disposable, and low-cost electrochemical biosensors.

**Figure 2:**
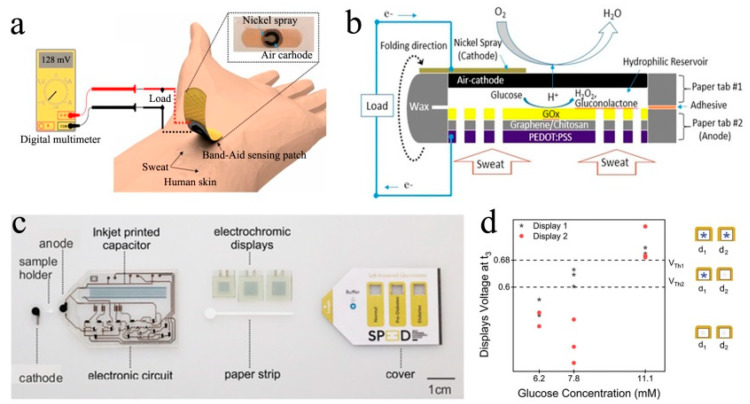
(**a**) Schematic diagram and (**b**) the sectional view of the paper-based EBFC-SPB for sweat glucose sensing on skin [54].(**c**) The structure of the self-powered glucometer and (**d**) the voltage change of this sensor in the presence of 6.2 mM, 7.8 mM, and 11.1 mM glucose [36]. Copyright 2021 John Wiley and Sons.

Nevertheless, the fabrication cost of disposable μ-EBFC-SPBs must be reduced to realize their transitions from fabrication to application [51,52]. Taking this into consideration, Choi et al. created a three-dimensional origami paper-supported μ-EBFC-SPB for detecting glucose [53]. In this study, the authors designed an enzyme-free cathode, avoiding the usage of expensive biological enzyme. Additionally, the cost of this μ-EBFC-SPB was reduced to USD 0.15. In addition, the sensitivity of this disposable sensor was also impressive (0.02 μA mM^−1^) due to the high activity of the screen-printed glucose oxidase anode. Shortly afterwards, this group reported a wearable μ-EBFC-SPB for monitoring blood sugar during exercise, taking advantage of the low-cost air cathode [54]. They used the electron-conductive, microporous, and easy processing graphene-doped PEDOT:PSS as the microfluidic reservoir. This flexible reservoir could boost the electron connection between the enzyme and the electrode, accelerate the mass transfer, and facilitate mass production of the μ-EBFC-SPB. The authors integrated this 3D-μ-EBFC-SPB with a Band-Aid adhesive patch, obtaining a wearable and on-site electrochemical biosensor (Figure 2a,b). This sensor patch could monitor the glucose levels in human sweat in vivo and exhibited a high sensitivity of 1.35 µA mM^−1^. Taken together, the above reports powerfully push forward the development of disposable μ-EBFC-SPBs in portable, easy-to-use, and personalized health monitoring devices. 

It is notable, however, that the reported single-use μ-EBFC-SPBs showed glucose detection ranges from 0.10 to 5.55 mM due to the low battery output caused by the low enzyme loadings on electrodes. The glucose concentration of 8 mM is the critical threshold that separates healthy people from hyperglycemic patients [55,56]. Therefore, the applications of the reported μ-EBFC-SPBs in health monitoring and management are problematic. In this context, Sabaté et al. proposed a novel signal amplification strategy by connecting the μ-EBFC-SPB with a capacitor in parallel [36]. The lower electrical energy produced by the μ-EBFC-SPB from analytes could be stored in the capacitor and released at a higher level. The voltage of the capacitor was directly related to the analyte concentration as the analyte was the only electron source. In particular, the authors designed an electrochromic display to discriminate the voltage area of the capacitor to indicate the analyte concentration (Figure 2c). Taking glucose as a model target, Sabaté et al. engineered a FAD-GDH/Ag_x_O biofuel-powered microfluidic biosensor. The authors tuned the voltage output of this integrated sensing system to selectively trigger the displays to turn on by choosing the capacitor with the right capacitance of 2 mF. In the presence of 6.2 mM, 7.8 mM, and 11.1 mM glucose, the voltages that could be applied to the displays were below 0.6 V, above 0.6 V but below 0.68 V, and surpassing 0.68 V, respectively. Additionally, the threshold voltages that triggered display 1 and display 2 to turn on were 0.6 V and 0.68 V (Figure 2d). Therefore, this integrated sensing device could discriminate between healthy (<7.8 mM), pre-diabetes (>7.8 mM), and diabetes (>11.1 mM) individuals, according to the working states of the displays. Additionally, this integrated μ-EBFC-SPB was expected to develop into a point-of-care health-monitoring device to indicate other health parameters by selecting suitable enzymatic catalysts. 

## 4. Wearable Microfluidic EBFC-SPBs

**Figure 3:**
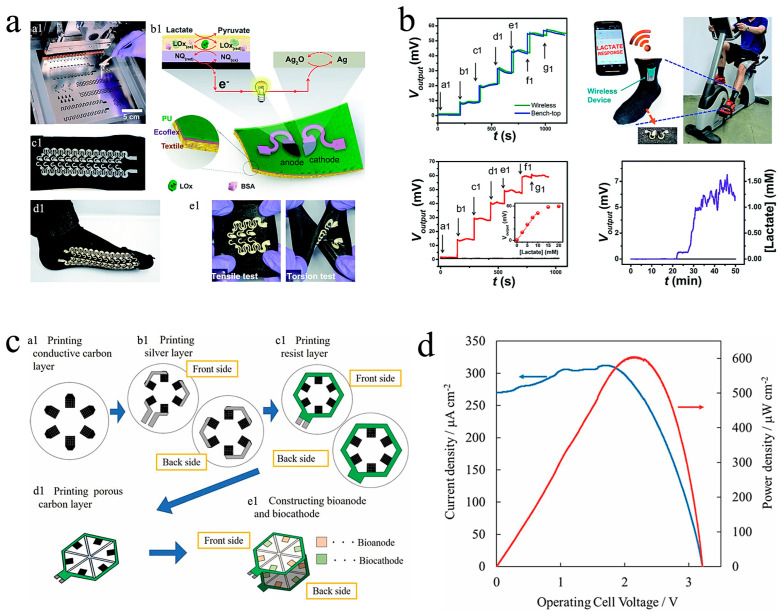
(**a**) Digital photograph and working principle of the wearable μ-EBFC-SPB fabricated using screen-printing technology; (**b**) Sensor’s flexibility tests and the ability of this sock-based biosensor to monitor lactate online [57] Copyright 2016 Royal Society of Chemisty. (**c**) Schematic diagrams of the screen-printed EBFC array; (**d**) the output performance of this EBFC array [58]. Copyright 2019 Electrochemical Society.

Lightweight, flexible and wearable electrochemical biosensors are highly desired for personalized medical devices. These wearable biosensors must be able to be integrated with the human body as well as provide continuous and timely signals. The essential challenges in wearable electrochemical sensors are miniaturization, wireless data transmission, and highly integrated devices since the traditional electrochemical sensors necessitate an external power supply device and a signal collector. In contrast, the EBFC-SPB is a new type of alternative electrochemical sensor due to their merits of self-sustaining power and ease of miniaturization. However, no wearable EBFC-SPBs were reported in the 15 years since the first EBFC-SPB was proposed, limited by the immature electrode engineering technology. In 2016, Wang’s group created the first wearable self-powered biosensor using screen-printing technology [57]. The mixtures of COOH-CNTs/mineral oil and COOH-CNTs/Ecoflex^®^/Ag/AgCl were used as the anodic and cathodic inks. Additionally, the inks were printed onto highly stretchable and wearable fabrics. After that, lactate oxidase or glucose oxidase was fixed on the cathode. Finally, a membraneless and wearable μ-EBFC-SPB was fabricated (Figure 3a). Due to the synergistic effects of nanomaterial-based engineered inks and the serpentine designs, the devised EBFCs could withstand severe mechanical deformations and maintained stable power output after 100 cycles. Significantly, the detection ranges of this wearable μ-EBFC-SPB for glucose and lactate were broadened to 0–50 mM and 0–20 mM, respectively. In addition, the glucose and lactate detection limits decreased to 6.71 ± 0.90 μW cm^−2^ mM^−1^ and 3.14 ± 0.20 μW cm^−2^ mM^−1^, respectively. The practical application of this flexible μ-EBFC-SPB in monitoring the health data in vivo was confirmed by integrating it with socks and a wireless device. The lactate produced by the wearer could be detected by this wireless and wearable μ-EBFC-SPB (Figure 3b). This study was expected to advance the application of μ-EBFC-SPBs in designing a non-invasive and wearable self-powered sensing device. 

However, differing from the requirements of disposable μ-EBFC-SPBs, those designing wearable μ-EBFC-SPB should pay more attention to the following aspects: (a) improving the output of the sensing system to drive the wireless signal transmission device, (b) increasing the stability of the sensing system in body fluids to extend the sensor’s life, and (c) shortening the sensor’s response time to obtain timely and online signals. For the wearable μ-EBFC-SPB created by Wang’s group, its voltage output was as low as 0.4 V. The sensor’s response time, in particular, was as long as 35 min on the volunteer’s body. In addition, the durability of this sensor was unclear. In this case, Tsujimura et al. fabricated a six-glucose/O_2_ BFCs-powered sensor using screen-printing technology (Figure 3c) [58]. This sensor had an output voltage of up to 3.2 V, which was expected to drive the wireless signal conversion device (Figure 3d). In addition, the limitation related to the O_2_ cathode on the sensor’s performance was eliminated by printing the MgO-templated carbon on water-repellent paper. The resultant glucose detection range of this μ-EBFC-SPB was from 1 mM to 25 mM, which covered the normal and abnormal glucose expression levels. Nevertheless, the power output sharply decreased when operating this μ-EBFC-SPB in artificial urine. 

**Figure 4:**
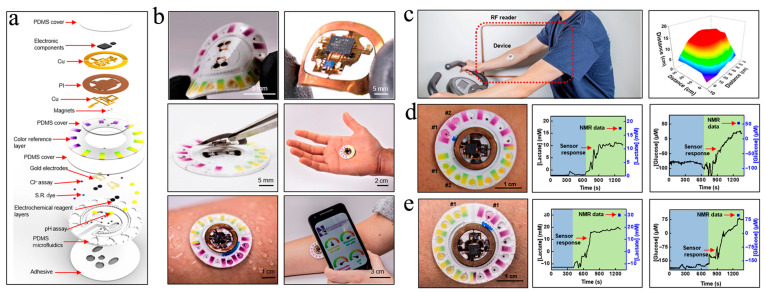
Schematic diagram of the components (**a**) and the structure (**b**) of this wearable and wireless μ-EBFC-SPB. PI: polyimide; S.R.: sweat rate. (**c**) The performance test of this wearable and wireless μ-EBFC-SPB on human skin, and its capacity in monitoring glucose (**d**) and lactic acid (**e**) in sweat on the human skin surface [59].

In light of this, Rogers et al. fabricated a lightweight, soft, and skin-interfaced μ-EBFC-SPB through the combined use of microfluidic technology and lithography technology [59]. They engineered exquisite channels, which could form a boosted interface with eccrine glands but avoid direct contact with skin interface. Consequently, the wearable μ-EBFC-SPB, driven by the pressure generated by the eccrine glands, could extract sweat from the skin surface without direct contact with the skin, perfectly avoiding cross-contamination (Figure 4a,b). In addition, these devices could robustly attach to the skin without failure during exercise. To improve the wearability of the sensor, the authors combined the sensing system with an electrochromic display to develop a wireless, visual, and wearable μ-EBFC-SPB. Taken together, this highly integrated demonstrator sensor could monitor the comprehensive health big data, such as the expressed levels of chloride, lactate, glucose, and protons in sweat, providing an in-depth indication of the volunteer’s health status. Furthermore, the sensor’s in vivo operating time was extended to 2 days due to the absence of physical damage and cross-contamination. In particular, its response time was shortened to 12 min despite using visualized electro-chromism as the signal output (Figure 4c–e). This study provides a big push to accelerate the implementation of wearable μ-EBFC-SPBs in continuous health monitoring. 

**Figure 5:**
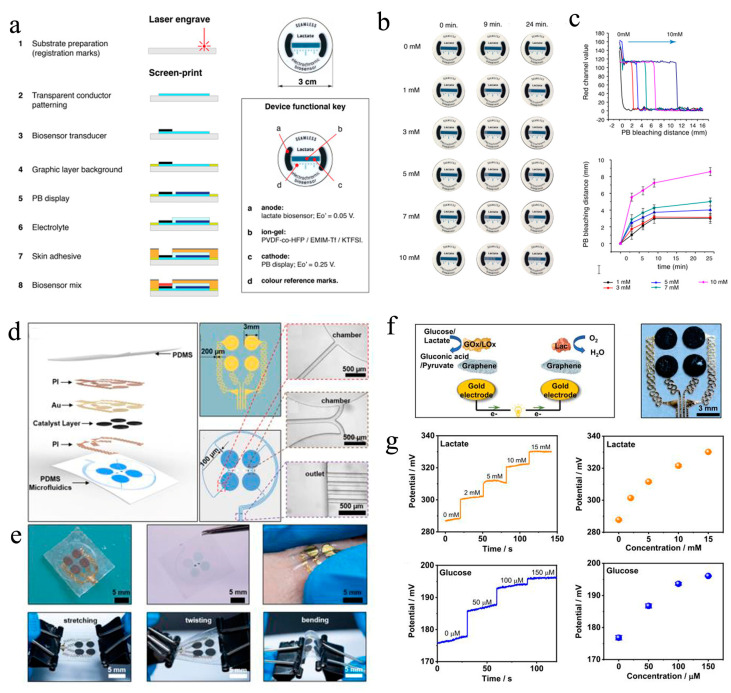
(**a**) Diagrammatic representation of the fabrication process and photographs of this paper-based μ-EBFC-SPB. The illustration shows the functions of the different components of this paper-based μ-EBFC-SPB; (**b**) evolution of the display color change in the presence of different lactate concentrations; (**c**) the PB bleaching distance and time response for lactate [60]. Copyright 2021 Elsevier. (**d**) Diagrammatic representation and (**e**) digital photographs of the configuration of the epidermal and stretchable μ-EBFC-SPB; (**f**) the working principle andthe enlarged photo of this epidermal μ-EBFC-SPB; (g) the sensing performance of thisμ-EBFC-SPB on skin surface[61].

In terms of the cost, most of the reported wearable μ-EBFC-SPBs rely on silicon-based chips, which complicate the fabrication process and increase the fabrication cost. To overcome this limitation, Campo et al. created a wearable and sustainable sensing device by printing the lactate oxidase/osmium-polymer anode and Prussian Blue (PB) display cathode on the flexible and transparent polymer of PEDOT:PSS (Figure 5a). Additionally, a gelling agent, VDF-co-HFP, and an ionic liquid, EMIM-Tf, were used to protect the PB cathode from bodily fluids and separate it from the anode. The low-cost, easy-to-process polymer materials were expected to enable the mass production of wearable μ-EBFC-SPBs (Figure 5b, c). Additionally, the length of the electrochromic display showed a linear relationship with the lactate in a concentration range of 0–10 mM. However, the presence of a diaphragm between the cathode and anode slowed down the electron/mass transfer rate. Eventually, the full response time was extended to 24 min. In addition, the authors verified the feasibility of this demonstrator device only in a buffer solution [60].

Shortly afterwards, Yu et al. designed an epidermal self-powered biosensor by designing a microfluidic system in an ultra-thin soft flexible polymer using electron-beam evaporation technology and photolithography technology [61]. Additionally, they used lactate oxidase/glucose oxidase and laccase as the anodic catalyst and cathodic catalyst, respectively (Figure 5d, e). Thanks to the highly specific catalysis of bio-enzymes, this μ-EBFC-SPB was designed as a membraneless device. The ultra-thin soft flexible polymer enabled the skin patch to effectively extract sweat and to withstand severe physical deformation. Additionally, the membrane-free design reduced the internal resistance of the EBFC, and the detection sensitivities of the sensor for lactate and glucose were thus further improved to 2.48 mV mM^−1^ and 0.11 mV μM^−1^, respectively. Additionally, this ultra-thin and flexible μ-EBFC-SPB could stick to any location on the body and generate real-time and in situ signals related to glucose and lactate (Figure 5f, g). Taken together, this study opens a new chapter in designing practical and wearable healthcare monitoring devices. 

**Figure 6:**
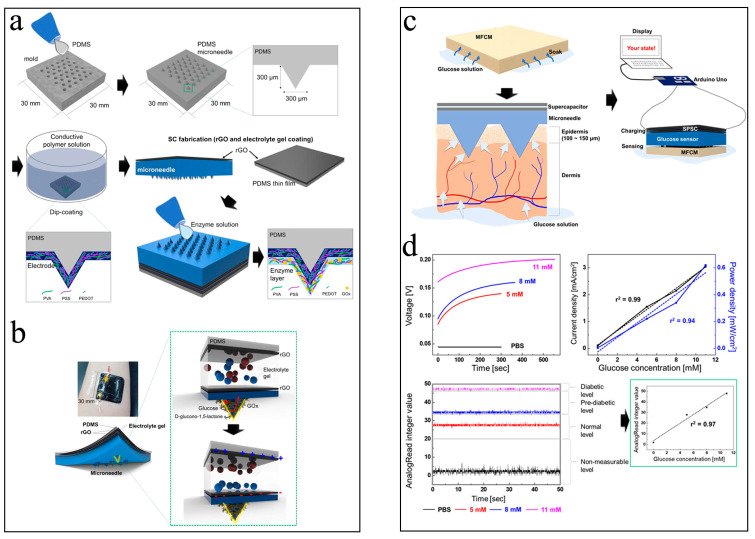
(**a**) Schematic diagram of the fabrication process of microneedle-type glucose sensor; (**b**) the sketch of composition of this microneedle-type glucose sensor; the working principle (**c**) and sensing performance tests of this glucose sensor (**d**) [47]. Copyright 2022 John Wiley and Sons.

Nevertheless, special attention is required regarding the fact that most of the reported μ-EBFC-SPBs were designed as skin patches to detect health related substances in sweat. This means that volunteers must produce enough sweat through exercise, etc. However, for sick populations, physical activity will pose extra detrimental effects to their bodies. Taking this into consideration, Park et al. fabricated a microneedle-type glucose sensing patch by connecting a microneedle glucose oxidase anode with a supercapacitor (Figure 6a, b) [47]. This sensing anode could penetrate deep into the surface layer of the skin, and could use the glucose in the interstitial fluids as the anodic fuels. Ultimately, this microneedle self-powered device was able to monitor the glucose in interstitial fluids without causing pain and inflammatory reactions. It is also worth noting that the electrons from the glucose oxidation on the anode could be stored in the capacitor, enabling the self-charging and capacitor-type EBFC-SPB to offer a high power density of 0.62 mW cm^−2^ that exceeded that produced by the glucose biofuel cell. In particular, in this study, an Arduino Uno board and programming software were used to process the output signals (Figure 6c, d). Thanks to the above advantages, this microneedle- and capacitor-type sensing device was able to distinguish normal, pre-diabetic, and diabetic glucose levels in a skin model.

**Figure 7:**
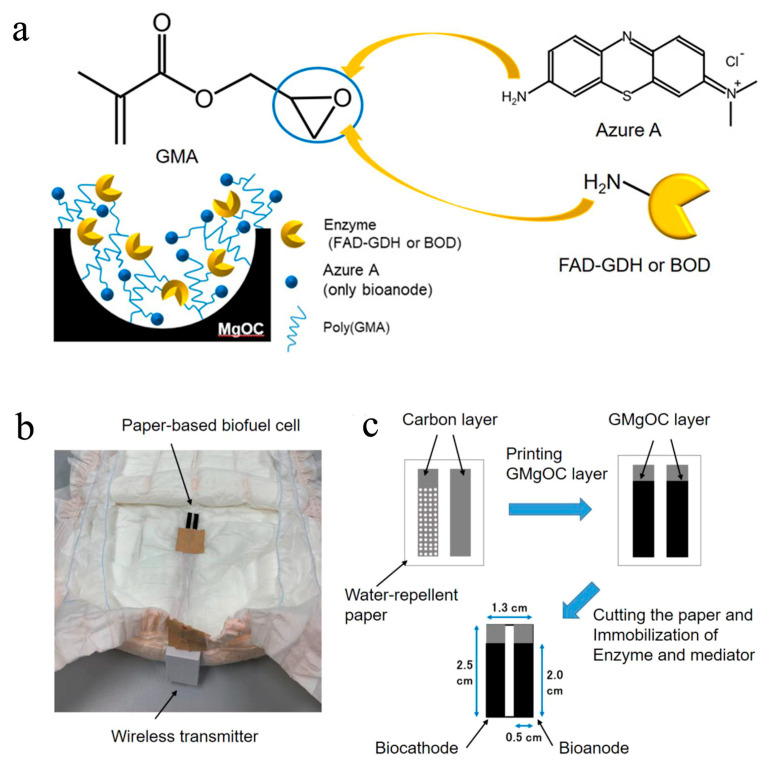
(**a**) Schematic diagram of the MgO-templated electrode in the diaper sensor. The FAD-dependent glucose dehydrogenase (FAD-GDH) and azure A were used as the anodic catalyst and electron-mediator, respectively; Digital photo of the self-powered diaper sensor connected to a wireless transmitter (**b**) and its fabrication process (**c**) [48]. Copyright 2021 American Chemical Society.

In addition to sweat and interstitial fluids, urine, as the study of Tsujimura et al. demonstrated, also carries a variety of non-invasive and informative biomarkers. Therefore, Tsujimura et al. reported a six-biofuel-powered sensing device to detect the glucose in urine in 2019. Unfortunately, this device was unable to analyze the actual urine sample, and the wearable applications had not yet been implemented [58]. To address the above issues, this group advanced the sensor’s design and created a diaper biosensor. In order to fulfill the goal of installing in diapers, the EBFC-SPB must be small and flexible. Given this, Tsujimura et al. used the well-printed MgO-templated carbon-coated paper as the supporting electrode. In addition, instead of using the flowing enzyme solutions, the enzymatic catalysts (FAD-GDH and BOD) and mediators (Azure A) were covalently bound to the electrode surface (Figure 7a) [48]. With the above improvements, the sensor’s size was reduced and the flexibility was improved. However, the open circuit voltage and maximum power density of this EBFC-SPB in 11 mM glucose were relatively low (0.77 V, 0.12 mW cm^−2^). Given this, the authors integrated this EBFC-SPB with a low-power signal transmission device, creating a wireless and wearable diaper sensor. The demonstrator device could detect the urine glucose in the range of 0−10 mM with a sensitivity of 0.0030 ± 0.0002 Hz mmol^−1^ dm^3^ (Figure 7b, c)). This study opened a new chapter in designing wearable urine sensors. Unfortunately, the choices of external signal transmission devices are limited due to the low power output of EBFC.

**Figure 8:**
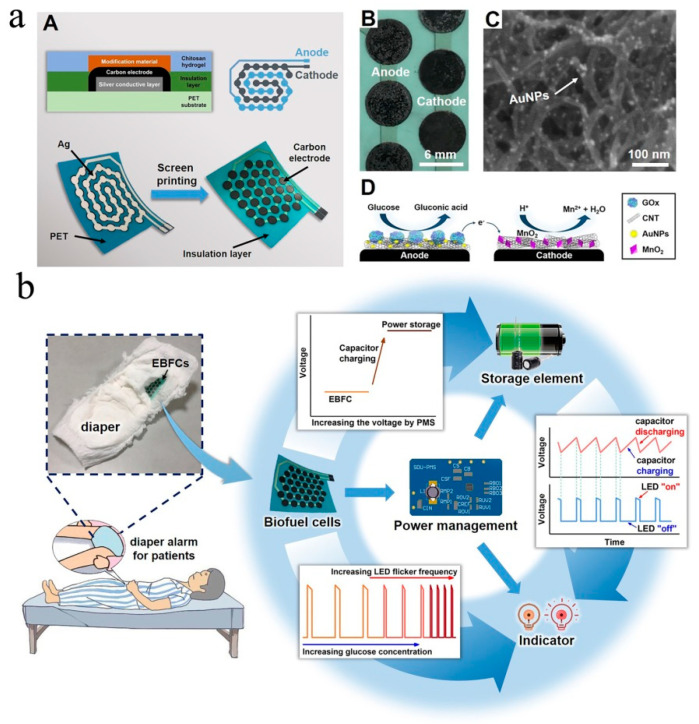
(**a**) Schematic diagram (**A**), electrode material characterization (**B**, **C**), and the working principle of the diaper μ-EBFC-SPB (**D**); (**b**) detailed view of the components of the diaper alarm and the circuit diagram of alarm device [62]. Copyright 2021 Elsevier.

To further improve the power output of the EBFC-SPB, Jiru Zhang et al. made three new improvements on the sensor’s design: designing winding electrodes on a flexible substrate using screen-printing technology, using carbon nanotubes/gold nanoparticles hybrids as the substrate material to accelerate the electron transfer between the enzymatic catalysts and electrodes, and integrating the sensor with a micro-capacitor to store and convert the electric energy generated by the EBFC (Figure 8a) [62]. Intriguingly, the winding electrodes significantly increased the electrode’s effective surface area on the substrate, and thus the power output of the EBFC was expected to improve. In addition, the micro-capacitor could store the relatively low electrical energy and release it instantly with higher power. Benefiting from the above improvements, the power density of the EBFC in 5 mM glucose reached 220 μW cm^−2^. The power of this EBFC was high enough to drive a light-emitting diode (LED), which flashed. Finally, the authors manufactured a diaper sensor with a simple structure and visual signal via integrating the EBFC-SPB with a small LED. This diaper sensor could quantificationally indicate the urine sugar concentration of the wearer via the LED’s flicker frequency. Additionally of note was the highly specific catalysis of enzymes, which endowed this EBFC-SPB with good anti-interference ability (Figure 8b). Taken together, this study powerfully progresses the practical process of wearable online health monitoring devices. 

## 5. Others

**Figure 9:**
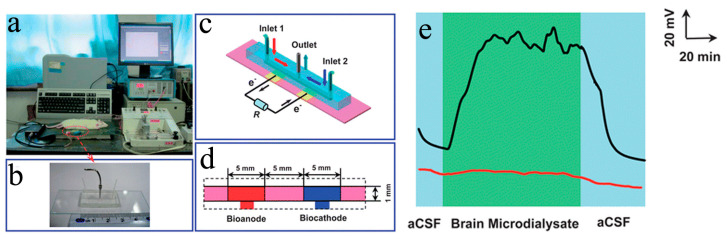
(**a**) Schematic diagram of the μ-EBFC-SPB for the continuous monitoring of the glucose in rat brain, the digital photograph (**b**) and schematic diagram (**c**,**d**) of this μ-EBFC-SPB. (**e**) The voltage-time response of the sensor recorded by continuously analyzing the cerebral neurodialysis fluid [63]. Copyright 2013 Royal Society of Chemisty.

The main applications of the reported disposable and wearable μ-EBFC-SPBs included detecting small molecules in the body fluids secreted or extracted from the body (sweat, urine, and blood). In 2013, Mao’s group created the first EBFC-SPB that could continuously monitor the neurochemicals in rat brains in vivo [63]. This sensing system consisted of two parts: an EBFC-SPB and an in vivo microdialysis system. To analyze the glucose in the cerebral neurodialysis fluid, they fabricated a glucose/O_2_ biofuel cell-powered biosensor using the microfluidic technique. Glucose dehydrogenase (GDH) and laccase were used as the anodic and cathodic catalysts, respectively. The microfluidic technique built a co-laminar between the anolyte and catholyte, enabling the GDH anode and laccase cathode to work independently of each other. The μ-EBFC-SPB was able to analyze the glucose quantitatively in vitro in a range of 0.2–1.0 mM. To realize the in vivo monitoring, they exported the cerebral nerve fluid from the rat brain and then directly introduced it to the microfluidic sensor using microdialysis technology (Figure 9a-d). The experiment’s results indicated that this integrated sensing system could continuously monitor the neurochemicals of glucose for 60 min (Figure 9e). This work proposed a prototype of a μ-EBFC-SPB for in situ monitoring and exhibited great application potential in the future medical monitoring field. 

**Figure 10:**
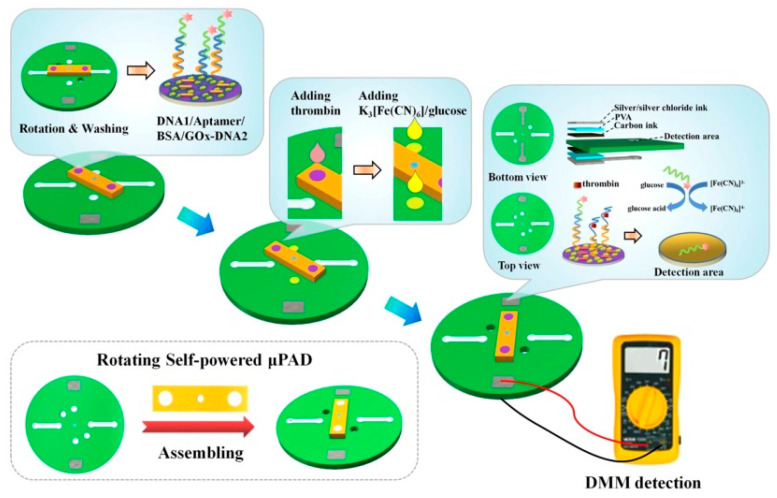
Schematic diagram of the rotational μ-EBFC-SPB and its working principle for thrombin detection [64]. Copyright 2022 Elsevier.

To realize the monitoring function of microfluidic self-powered biosensors in disease markers, Chen’s group constructed an advanced microfluidic self-powered biosensor with a rotating frame to detect thrombin [64]. As Figure 10 depicts, this rotating self-powered sensor included three parts: a detection area, the super-capacitor, washing channels, and the hollow holes. In the detection area, the potassium ferricyanide worked as the cathode reactant, and the anode was modified with a sandwich structure of DNA1-thrombin aptamer-DNA2/GOD. The super-capacitor was used to store and amplify the electric signal. The thrombin could be firmly captured by its aptamer and further trigger the shedding of GOD from the anode. The free GOD could catalyze the glucose oxidation and form a complete battery pathway with the reduction of potassium ferricyanide at the cathode. The supercapacitor could be charged by this battery and then output an electrical signal that is linear with the thrombin concentration. It is also worth noting that the processes of reaction, incubation, and washing did not interfere with each other by rotating the reaction disc. The above ingenious design endowed this microfluidic self-powered biosensor with outstanding sensing performance in a linear range of 3–150 nM with a low detection limit of 0.9 nM. In particular, by replacing appropriate aptamers, this sensing system could be used to detect other disease markers, such as proteins and nucleic acid, etc. This work lays a solid foundation for the application of microfluidic self-powered biosensors in portable point-of-care testing.

## 6. Conclusions and Perspectives

EBFC-SPBs have been considered ideal portable biosensors since they were proposed due to the advantages of self-supported power, simple structure, and high specificity. However, its miniaturization is plagued by the following factors: the cathodic reaction is disturbed by the perturbation of the anodic fuel near the cathode surface and vice versa, and the output of the EBFC decreases as its size decreases. Since the EBFCs in the self-powered bio-sensing systems serve as the energy driver and the signal generator, the sensor’s performance is thus inseparable from that of the EBFCs. Specifically, the service life of the EBFC-SPBs is directly determined by the long-term stability of the EBFC in the biological fluids. Additionally, the EBFCs with high electricity output (voltage, current, and power density) endow the EBFC-SPBs with high sensitivity. Significantly, the microfluidic and screen-printing technologies have brought new opportunities for the development of miniaturized, portable EBFC-SPBs. The exquisite fluid channel designed using microfluidic technology enables the formation of a stable co-laminar between the anolyte and catholyte, thereby avoiding the mutual interference between the two electrode reactions. The screen-printing technology can print the electrodes on a variety of substrate surfaces, reduce the cost, and improve the flexibility of the EBFC-SPB. The combination of these two technologies not only improves miniaturized EBFC-SPBs’ output and sensitivity, but, more importantly, promotes the development of integrated EBFC-SPBs. Thanks to the microfluidic and screen-printing technologies, disposable and wearable EBFC-SPBs have been developed for online health monitoring. Nevertheless, the further development of μ-EBFC-SPBs in practical application still faces challenges.

For the reported disposable μ-EBFC-SPBs, continuous efforts have been devoted to reducing sensor cost and improving sensor sensitivity. It is through decades of hard work that the cost of disposable μ-EBFC-SPBs has decreased to a relatively low level of approximately USD 0.15, and their sensitivities are high enough to distinguish between healthy and unhealthy states. However, aside from the cost and sensitivity, attention should be focused on the reprocessing of disposable μ-EBFC-SPBs. If the metal-based or polymer-based electrode materials are handled clumsily, this will cause huge economic losses and environmental pollution. Therefore, future efforts should be devoted to efficiently recycling metal-based electrode materials and designing eco-friendly or degradable polymer electrodes. 

Unlike disposable μ-EBFC-SPBs, in addition to sensitivity, the response time and long-term stability are also essential factors for wearable μ-EBFC-SPBs. With persistent efforts, the sensitivities of wearable μ-EBFC-SPBs were significantly improved. Furthermore, their response time has been reduced from 20 min to 4.6 s, which is fast enough to meet the requirements of online monitoring. Unfortunately, the long-term stability of the wearable μ-EBFC-SPBs was overlooked. The main reasons are that the natural enzymes are inherently fragile and the complex bio-fluids cause additional damage to the enzymes. Therefore, to accelerate the implementation of the stable, continuous, online monitoring function of the wearable μ-EBFC-SPBs, it should be the focus of future work to solve the problems affecting the sensor’s long-term stability. Toward this end, we think the nanozymes, which possess enzyme-like catalysis but outstanding catalytic stability far exceeding that of natural enzymes, will be an ideal alternative electrocatalyst for enzymes in designing stable, wearable self-powered biosensors [65,66,67]. In addition, it is also an effective way to develop an enzyme protection strategy to protect enzymes from being attacked by the bio-fluids. In 2022, Zhu’s group encapsulated the nanowire of SWCNT, as well as the enzymatic catalysts of GOD and horseradish peroxidase (HRP) into the hydrophilic MAF-7 using in situ embedding technology. The obtained biocatalyst (SWCNT-MAF-7-GOx/HRP) showed highly stable glucose oxidation activity in a harsh environment. In particular, the EBFC catalyzed by the SWCNT-MAF-7-GOx/HRP exhibited an eightfold increase in power density and a 13-fold increase in stability in human whole blood compared with the EBFC catalyzed by the unprotected enzyme [8]. This study suggested that the requisites for protecting enzymatic electro-catalysts are: to create a protective layer on the enzyme surface and to ensure the fast mass transfer rate and fast electron transfer rate.

Overall, despite the existing problems and challenges, portable, low-cost, and miniaturized μ-EBFC-SPBs have already achieved considerable progress in online health monitoring. Additionally, we believe the μ-EBFC-SPBs will realize their practical application and powerfully push the development of future healthcare since the problems and challenges are widely identified, and the unprecedented development of electrode design and fabrication technologies will help us find effective solutions.

## Data Availability

Not applicable.
